# Foamy Virus Assembly with Emphasis on Pol Encapsidation

**DOI:** 10.3390/v5030886

**Published:** 2013-03-20

**Authors:** Eun-Gyung Lee, Carolyn R. Stenbak, Maxine L. Linial

**Affiliations:** 1 Fred Hutchinson Cancer Research Center, Basic Sciences Division; 1100 Fairview Avenue North, Seattle, WA 98109, USA; E-mails: elee@fhcrc.org (EGL); mlinial@fhcrc.org (MLL); 2 Seattle University, Biology Department; 901 12th Avenue, Seattle, WA 98122, USA; E-mail: stenbakc@seattleu.edu

**Keywords:** Foamy virus assembly, Pol expression, Pol encapsidation, Pol enzymatic activities

## Abstract

Foamy viruses (FVs) differ from all other genera of retroviruses (orthoretroviruses) in many aspects of viral replication. In this review, we discuss FV assembly, with special emphasis on Pol incorporation. FV assembly takes place intracellularly, near the pericentriolar region, at a site similar to that used by betaretroviruses. The regions of Gag, Pol and genomic RNA required for viral assembly are described. In contrast to orthoretroviral Pol, which is synthesized as a Gag-Pol fusion protein and packaged through Gag-Gag interactions, FV Pol is synthesized from a spliced mRNA lacking all Gag sequences. Thus, encapsidation of FV Pol requires a different mechanism. We detail how WT Pol lacking Gag sequences is incorporated into virus particles. In addition, a mutant in which Pol is expressed as an orthoretroviral-like Gag-Pol fusion protein is discussed. We also discuss temporal regulation of the protease, reverse transcriptase and integrase activities of WT FV Pol.

## 1. Introduction

Foamy viruses (FVs) are ancient and highly successful retroviruses. The integration of retroviral genomes creates endogenous retroviral elements that have been left as a fossil record in host species. Although rare, cases of endogenous foamy viruses have been reported in the genomes of sloths [1] and the aye-aye, a strepsirrhine primate from Madagascar [2], and recently, an endogenous foamy virus-like element was discovered in the coelacanth genome [3]. These findings extend the history of coevolution between FV and their hosts to more than 400 million years, identifying it as the most ancient of all retroviruses. FVs are complex retroviruses that infect most non-human primates (NHP), cattle, cats and horses (reviewed in [4]). In contrast to complex orthoretroviruses, which are sometimes highly pathogenic, FVs establish persistent infections in the absence of pathogenicity. Despite this lack of pathogenicity, FVs are readily transmitted within host species. There are no reports of human-specific FVs. However, zoonotic transmission from NHP to humans has been documented in various contexts, including natural habitats and occupational exposures. FV transmission to humans provides the potential for the emergence of new strains of FV that could pose a risk to humans [5,6]. 

Despite the fact that the FV genomic organization is similar to that of orthoretroviruses, FV replication differs in many ways, and as such, they comprise the only genus of the retroviral subfamily, *Spumaretroviridae*. FVs reverse transcribe their encapsidated RNA genome during assembly and/or budding, leading to the production of DNA containing virions. FVs package RNA; but, infectious virions contain double-stranded DNA (dsDNA), and in this way, FVs resemble hepadnaviruses, such as hepatitis B virus (HBV), whose genomic organization differs from that of retroviruses. Thus, FVs bridge the gap between retroviruses and hepadnaviruses (reviewed in [7,8]). The focus of this review is the process of FV assembly, with emphasis on Pol encapsidation, which occurs by a mechanism different from both orthoretroviruses and hepadnaviruses. 

**Figure 1:**
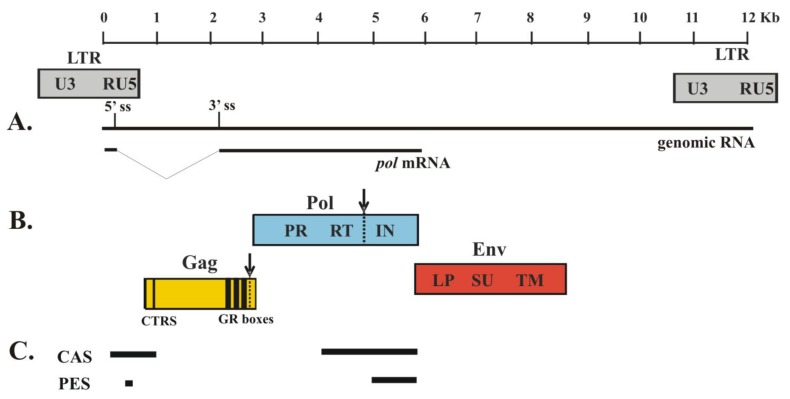
Genome of prototype foamy virus (PFV). Depicted is the molecular clone PFV-13 (GenBank accession no. U21247; 11,954 bases). The shaded boxes below the genome indicate the location of the LTR promoter regions in the proviral DNA. (**A**) The thin lines represent the genomic RNA and mRNA for Pol protein. Pol is expressed from a spliced mRNA. The 5´ splice site (ss) and the 3´ splice site (3´ss) are indicated. (**B**) The colored boxes indicate the three major PFV protein products: Gag, Pol and Env. PR-mediated cleavage sites are indicated with dashed lines and arrows. CTRS, cytoplasmic targeting and retention signal; GR boxes, glycine/arginine-rich motifs; PR, protease; RT, reverse transcriptase; IN, integrase. LP, leader peptide; SU, surface domain; TM, transmembrane domain. (**C**) The thick lines represent RNA sequences required for proper assembly. *Cis*-acting sequences (CAS) are required for genomic RNA packaging; Pol encapsidation sequences (PES) are required for Pol packaging.

Retroviral assembly requires coordinated packaging of genomic RNA and viral proteins. In orthoretroviruses, packaging sequences are located near the 5´ end of genomic RNA (termed ψ) and are specifically recognized and bound by the nucleocapsid (NC) domain of Gag, which contains highly conserved cysteine-histidine (CH) motifs flanked by basic residues (reviewed in [9]). In alpharetroviruses, such as avian sarcoma leukosis virus (ASLV), ψ is located upstream of the 5´ splice site (ss), resulting in the inclusion of ψ in both unspliced genomic RNA and spliced *env* RNA, and there is an undefined mechanism to exclude spliced RNA from virions (reviewed in [9]). In contrast to orthoretroviruses, FV *cis*-acting sequences (CAS) for genome packaging are located at several sites in the genome (Figure 1). CAS I is located in the 5´ untranslated region (UTR) and CAS II is in the 3´ end of the *pol* gene. The details of how the two CAS elements function in a concerted manner for genome packaging remain unknown. Since the sequences of CAS II, which were found to be important for genome packaging [10], are located in both subgenomic *pol* mRNA and genomic RNA, CAS I might be required and, thus, prevent *pol* mRNA from being packaged. FV Gag does not contain CH motifs, but there are two or three copies of a glycine/arginine-rich motif (GR box) near the C-terminus (Figure 1). GR boxes are thought to be functionally equivalent to CH motifs. GR box 1 has nucleic acid binding activity *in vitro*, whereas GR box 2 contains a nuclear localization signal [11,12]. Even when present, there is no known function for GR box 3. Overall, GR boxes are required for genomic RNA encapsidation and also play important roles in Pol incorporation, reverse transcription, virion morphology and infectivity [13,14,15]. 

The intracellular assembly of FV is similar to that of the betaretroviruses (B/D type), such as Mason Pfizer monkey virus (MPMV), in that a cytoplasmic targeting-retention signal (CTRS) within the Gag protein (Figure 1) directs nascent Gag to a pericentriolar region of the cell, specifically the microtubule organizing center (MTOC), for capsid assembly [16]. Cellular proteins are known to be involved in viral assembly, including that of FV. In some viruses, proteins associated with cellular mRNA metabolism located within P bodies and stress granules have been implicated in viral replication and assembly (reviewed in [17]). For example, Mov10, a putative RNA helicase, inhibits HIV-1 replication at multiple stages, including reverse transcription and virus production [18,19]. DDX6 and DDX3, members of the DEAD-box RNA helicase family, are required for efficient replication of hepatitis C virus, a member of the family *Flaviviridae* [20,21]. After FV infection, DDX6 has been shown to be translocated from the P bodies and stress granules to the MTOC and is thought to play important roles in conformational rearrangement of FV genomic RNA to facilitate efficient encapsidation [22].

## 2. FV Pol Expression

One of the major differences between FVs and orthoretroviruses is the mode of Pol expression. In orthoretroviruses, Pol is synthesized as a Gag-Pol fusion protein through either suppression of translation termination at the C-terminus of Gag or ribosomal frameshifting from Gag into the Pol reading frame (reviewed in [9]). These events are infrequent and result in higher expression levels of Gag relative to Gag-Pol. Specific motifs within the orthoretroviral Gag protein are sufficient to direct capsid formation, and Gag-Pol is assembled into particles using these motifs. FV Pol expression is also different from that of the closely related hepadnaviruses. Hepadnaviruses use internal promoters to generate a series of mRNAs, which are translated separately to produce the individual gene products, including the polymerase protein [23]. FVs express Pol from a spliced mRNA generated using the same 5´ss as for the *env* mRNA and the 3´ss located within the *gag* gene, upstream of the Pol start codon (Figure 1) [24,25,26,27,28]. Regulation of FV Pol protein expression to achieve proper Gag:Pol ratios may occur through regulation of *pol* mRNA splicing, as FVs have been shown to use a suboptimal 3´ss [29]. Translation efficiency may also contribute to the regulation of Pol levels within the cell, but little is known about the levels of translation initiation for Pol relative to Gag. 

## 3. FV Pol Encapsidation

Expression of FV Pol independently of Gag requires a different mechanism for Pol incorporation into virions from that of orthoretroviruses. FV Pol expression is similar to that of HBV polymerase in the sense that it is expressed independently of the capsid protein, but their mechanisms for polymerase encapsidation are different. Incorporation of HBV Pol depends on the binding of Pol to the packaging sequence, epsilon, at the 5´ end of pregenomic RNA, and the RNA/Pol complex initiates capsid assembly (reviewed in [23]). In contrast, FV Pol is not required for either RNA packaging or capsid assembly [30]. 

Genomic RNA is required for encapsidation of the FV Pol protein. Two *cis*-acting sequences within the genomic RNA, called Pol encapsidation sequences (PES), have been shown to be required for FV Pol packaging (Figure 1) [10,31]. The PES map within the *cis*-acting sequences (CAS) that is essential for RNA packaging [10,32,33]. In FV assembly, PES and CAS are both located in the coding region of *pol*, making it difficult to distinguish between RNA packaging and Pol incorporation. To overcome this difficulty, a FV four-vector system has been developed in which Gag, Pol, Env and packageable RNA are expressed from separate constructs [10,34,35]. In the four-vector system, one component can be mutated without affecting the sequences of the other three. This system has the advantage of separating changes in viral proteins from changes in the genomic RNA. However, a potential problem of this system is that each component is overexpressed, so that the normal ratios of the viral components are not retained, and the results may not reflect interactions that occur during normal infection. 

Several mechanisms have been proposed for FV Pol encapsidation. One mechanism is that Pol directly binds to the genomic RNA concurrently with RNA packaging via Gag binding [10,31]. Thus, RNA could act as a bridge between Gag and Pol. Alternatively, it is also possible that Pol directly binds to Gag, and this complex binds to RNA via Gag sequences to allow Pol packaging. One approach to examine if a Gag-Pol interaction is required for Pol packaging is to identify Gag mutations that affect Pol packaging, but not RNA packaging. Mutations in the first GR box (GR1) of Gag do not prevent RNA packaging, but lead to a defect in Pol packaging [14]. It is the clustered basic residues in GR1 that are required for Pol packaging, suggesting that interactions between Gag and Pol are required for Pol encapsidation. The requirement of Gag for Pol encapsidation could involve transit of Pol to the MTOC, where Pol has been shown to colocalize with Gag [16,36]. A specific localization sequence, such as the CTRS found in Gag, has not been identified for Pol. Thus, an intriguing possibility is that Gag-Pol binding may be required to transport Pol to the site of capsid assembly. Another possible role for Gag in Pol packaging could be that Pol alone can bind to RNA through PES, but Gag binding stabilizes the Pol-RNA complex. Alternatively, it is Gag in the Gag-Pol complex that binds to RNA at PES. Having RNA binding specificity in Gag rather than Pol is consistent with the fact that Pol must traverse the entire length of genome to synthesize cDNA. We do not know where in the cell interactions between Gag and Pol take place. Since both proteins are transiently localized in the nucleus [12,37], it is possible that Pol interacts with Gag in the nucleus, and Gag-Pol complexes are then translocated to the capsid assembly site through the CTRS in Gag proteins, although it could be difficult for such a large protein complex to exit the nucleus. However, in opposition to this hypothesis, an FV Gag mutant lacking the nuclear localization signal in Gag GR2 has WT levels of Pol encapsidation [14]. 

As Pol incorporation requires binding to the PES in genomic RNA, the number of Pol molecules per virion would be limited. Each of the two copies of FV genomic RNA would accommodate one or at most a few Pol dimers. Surprisingly, quantification of Pol molecules within purified FV particles found that an approximate ratio of Gag to Pol is 16:1, which is a higher level of Pol than that predicted by the RNA-only model [38]. Alternatively, if a large Gag-Pol complex binds to RNA, it would be possible to incorporate many more Pol molecules into each virion. 

In orthoretroviruses, the Pol precursor (PrPol) is incorporated into virions as a Gag-Pol fusion protein and cleaved by protease into three components, protease (PR), reverse transcriptase/RNase H (RT) and integrase (IN). FV PrPol is cleaved only once between RT and IN, yielding two mature proteins, PR-RT and IN. Only FV PrPol, not the individual cleavage products, is incorporated into virus particles [10,39]. A Pol mutant lacking the IN domain was shown to be deficient in incorporating Pol into virions [39]. Further analysis using a series of IN truncation mutations in the context of a full-length proviral vector revealed that the C-terminus of IN is required for Pol packaging [40]. As the C-terminus of IN contains part of the PES required for Pol packaging, the same IN mutants were tested using the FV four-vector system. No Pol packaging was found in the IN truncation mutants, despite the ability of these mutants to package viral RNA. This result suggests that the IN protein contains either a PES-binding domain or a Gag-binding domain that allows Pol to associate with Gag for RNA binding. It remains to be determined how Gag and Pol proteins interact for Pol incorporation. 

## 4. FV Pol Enzymatic Activities

### 4.1. Protease

Orthoretroviral Gag is cleaved into at least four proteins, whereas FV PR only cleaves Gag once, releasing a 3 kD peptide (p3) from the C-terminus. As a result, FV particles never mature. Infectious FV resembles the immature orthoretroviral virions in the assembly stage prior to Gag cleavage. The single cleavages in Gag and Pol are absolutely required for FV infectivity and replication [41,42,43]. Like orthoretroviral PRs, FV PR is an aspartyl protease that is only active as a homodimer. There are two copies of an Asp-Ser/Thr-Gly triplet at the active site to which each monomer contributes one triplet [44,45]. While orthoretroviral PRs form stable dimers [46], FV PR forms a weak dimer that exhibits proteolytic activity *in vitro* only at NaCl concentrations of 2–3 M [47,48]. However, biochemical and biophysical evidence indicates that under physiological conditions, PRs of simian foamy virus from macaque (SFVmac) and prototype foamy virus (PFV) are predominantly monomers in solution with or without the RT domain present [49,50]. In addition, SFVmac PR homodimers can only be detected as minor transient species, constituting only a small fraction of the total protein [51]. Given the largely monomeric state of PR and PR-RT under physiological conditions, it is likely that FV PR requires additional viral and/or cellular factors for efficient dimerization *in vivo*. 

Various mechanisms have been proposed for the activation of FV PR. One proposed mechanism for PFV Pol dimerization is that a dimerization domain within IN is required [40]. As retroviral IN works as a dimer or higher-order complex for efficient integration of viral DNA into host genomic DNA [52], it is possible that the IN domain in FV PrPol is sufficient for PrPol dimerization. The solution structure of the PFV IN tetramer has been solved [53,54]. An IN truncation mutant lacking two thirds of the C-terminus of IN was shown to exhibit defective processing of Gag and Pol, as well as defective Pol packaging into virions [40]. Introduction of a leucine zipper dimerization motif downstream of the IN truncation restores PR activity in cells. However, Pol encapsidation is not rescued, suggesting that Pol dimerization is not sufficient for Pol encapsidation. While IN dimerization is required for PrPol dimerization and PR activity, integrase activity is not [39,40,55,56]. 

Another mechanism has been proposed for FV PR activation. It was shown that a specific protease-activating RNA motif (PARM) located within the PES in the *pol* region of genomic RNA stimulates PR activity of PR-RT proteins [55]. The distinct RNA structure of this region is thought to be responsible for binding to Pol, which allows Pol dimerization required for PR activation. This result suggests a unique mechanism for FV PR activation through a viral RNA sequence, PARM. It was also proposed that in the presence of PARM, PR is active independently of the IN domain [56], which conflicts with the studies described in the previous paragraph in which IN deletion mutants are defective for PR activity. In one study designed to circumvent the strict requirement of IN for Pol encapsidation, and therefore PR activity *in vivo*, a Gag-PR-RT fusion protein lacking IN was created and expressed [56]. In this experiment, the Gag-PR-RT fusion protein is incorporated into virions and exhibits PR activity in the absence of the IN domain, indicating that Gag can provide PR activation normally supplied by IN. A recent report showed that uncleaved PrPol is more efficient in Gag processing than the PR-RT cleaved subunit [57], supporting a role for IN in PR in wild-type infection. Characterization of the PR activation mechanism requires further investigation. 

### 4.2. Reverse Transcriptase

RTs have two enzymatic activities, polymerase and RNase H, which cleaves the RNA strand of an RNA-DNA duplex. These two enzymatic activities are both necessary and sufficient for RT to convert the single-stranded viral RNA genome into dsDNA (reviewed in [9]). While FV RT demonstrates both enzymatic activities, *in vitro* studies have revealed two major differences in polymerase activity relative to some orthoretroviral RTs. First, FV RT is more processive and more active on a variety of templates [58]. Second, FV RT has a lower affinity for dNTPs [50,59,60]. FV RT is also structurally different from orthoretroviral RTs in that it acts as part of a PR-RT polyprotein. It has been shown that FV RT retains activity in the presence of additional protein domains, as part of a PrPol protein [39] and as part of a Gag-Pol fusion protein [36,61]. 

Much effort has been devoted to determining the precise mechanism by which reverse transcription occurs, and models have been derived for both orthoretroviruses (reviewed in [9]) and hepadnaviruses (reviewed in [23,62]). One notable difference between the two mechanisms is the primer used to initiate first strand synthesis. Hepadnaviruses have an additional terminal protein (TP) domain in RT that serves as the primer for reverse transcription [63], while orthoretroviruses use a host cell tRNA as primer. FV RT lacks the hepadnaviral TP domain, and the primer binding site in FV genomic RNA is complementary to tRNA lys1,2. Thus, the mechanism of FV reverse transcription is thought to be similar to that of orthoretroviruses. However, a key difference between FV and orthoretroviral reverse transcription is the timing of this event within the lifecycle. Upon entering a new host cell, reverse transcription proceeds as an early event for orthoretroviruses. In contrast and reminiscent of hepadnaviruses, reverse transcription occurs primarily as a late event in the FV lifecycle during assembly and/or budding. Although there are reports of some reverse transcription early after infection [64,65], the completion of reverse transcription late in the lifecycle leads to the infectious genome being dsDNA [66,67]. An FV mutant in which Pol was expressed as an orthoretroviral-like Gag-Pol fusion protein showed that even in this context, FV RT remains active late in the lifecycle [68]. Thus, the timing of FV RT activation and reverse transcription is intrinsic to Pol sequences and is not dependent on the mode of Pol expression.

Other viral or cellular factors may be involved with RT activity during viral assembly. A recent study using a FV four-vector system found that Gag processing is required for initiation of reverse transcription [57]. It is not yet clear whether the precursor Gag protein somehow inhibits RT activity during assembly or whether the cleaved p3 peptide has a stimulatory effect on RT. It is also possible that cellular proteins can contribute to RT activation, as is the case for hepadnaviruses (reviewed in [62]).

### 4.3. Integrase

Integrase functions early in the viral lifecycle to integrate reverse-transcribed dsDNA into the cell genome, and this is also true for FVs. FV IN, like orthoretroviral INs, contains an N-terminal zinc finger domain, a critical aspartic acid in the active site and a DNA binding domain. Studies of purified FV IN demonstrated both endonuclease and integrase activities [69,70]. FV IN also contains a strong nuclear localization signal (NLS) within the C-terminal domain of the protein [71,72]. Orthoretroviral IN acts as part of a large subviral nucleoprotein complex, known as the pre-integration complex (PIC) (reviewed in [73]). In this context, IN is involved in the transport of the PIC to the nucleus of the infected cell, and recent evidence suggests that the same is true for FV IN [74]. 

Despite much effort to determine the crystal structure of retroviral IN proteins, to date, PFV IN is the only such protein for which high-resolution structures have been obtained. The recent studies of PFV IN in complex with viral DNA have provided structural insights into retroviral IN enzymes and specifically PFV IN structures [53,54,75]. Although initial studies found that PFV IN exists in a monomer-dimer equilibrium in solution [76], more recent work has shown that PFV IN exists exclusively as a monomer in solution, in the absence of DNA [54,77]. This is in contrast to HIV-1 IN, which exists in a tetramer-dimer equilibrium in the absence of DNA [76]. PFV IN does form dimers upon interaction with its dsDNA substrate, and these dimers subsequently interact to form tetramers [53,54]. Tetramer formation is required to bring the target DNAs together and to generate a functional IN active site [53,54]. It is currently unclear how the monomeric and higher order states of FV IN are controlled within the viral lifecycle, and the involvement of cellular factors, as is seen in some orthoretroviruses, cannot be ruled out (reviewed in [78]). 

### 4.4. Regulation of Pol Enzymatic Activities

In orthoretroviruses, such as HIV-1, the PR domain in the Gag-Pol fusion protein can form only a weak transient dimer, resulting in low PR activity, until it is assembled into virions [79,80,81]. After the virion assembles, PR is at a high local concentration, forms stable dimers and becomes active. Given that FV Pol is expressed independently of Gag, the regulation of FV PR activity is likely to be different from that of orthoretroviruses and is currently poorly understood. Coupling PR activation to Pol incorporation would restrict PR activity and prevent cleavages of Gag and Pol until virus assembly takes place. It has been hypothesized that FV Pol dimerizes during virus assembly for activation. For this reason, in many studies, when processing of Gag and Pol is found in the cellular supernatants, Pol has been thought to be encapsidated into virions [10,31,39,55]. However, the preponderance of evidence shows that FV PR can be activated intracellularly and processing of Gag and Pol can occur independently of capsid assembly and in the absence of Pol incorporation into particles. For example, PR-mediated cleavage can occur intracellularly when cells are transfected with a Gag CTRS mutant or even in the complete absence of Gag [31,39,82,83]. Also, mutants that fail to package Pol into particles, such as GR box 1 mutants and an FV four-vector system lacking the RNA vector, are shown to cleave Gag and Pol in the cell [14,56]. 

**Figure 2:**
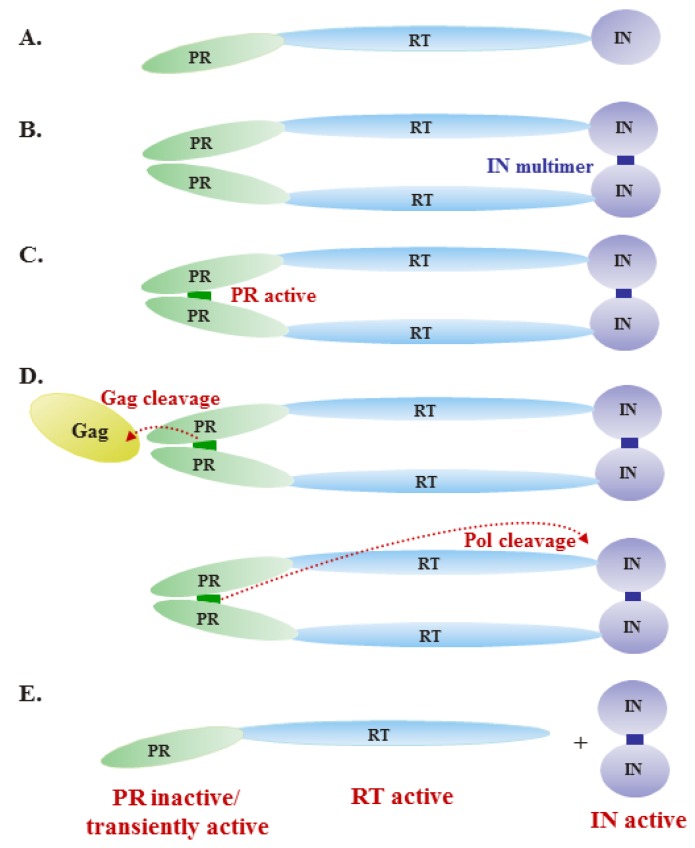
Model of regulation of Pol enzymatic activities during virus assembly. (**A**) The precursor Pol protein contains the protease (PR), reverse transcriptase (RT) and integrase (IN) domains. (**B**) Upon incorporation into virions, the precursor Pol protein forms dimers through IN-IN interactions. (**C**) The dimerization of precursor Pol allows formation of the PR active site. (**D**) Active PR cleaves Gag and Pol proteins, as indicated by the dashed lines. (**E**) After cleavage, PR-RT remains a monomer and is active as a polymerase, initiating reverse transcription of the viral RNA genome. Free IN is also active.

During normal FV infection, PR and RT are not cleaved from each other. This is the only known example of a protein that has both protease and nucleic acid polymerizing activity. Like orthoretroviruses, FV PR requires dimerization for activation. As described above, FV PR-RT exists predominantly as a monomer in solution and forms only transient dimers [49,51]. FV RT is active in this monomeric structure of the PR-RT [59]. Thus, the PR-RT molecule must adopt both monomeric (for RT) and dimeric (for PR) states, depending on the stage of the viral lifecycle and the enzymatic activity required. We propose a model of regulation of Pol enzymatic activities during viral assembly that takes into consideration this paradox (Figure 2). The precursor Pol protein is incorporated into virions and forms dimers by IN-IN interactions and/or through binding to PARM in genomic RNA. The dimerization of PrPol creates a PR active site, leading to cleavage of Gag and Pol. Although the exact order of the Gag and Pol cleavages is unknown, it is likely that Gag processing precedes Pol processing. After cleavage, PR-RT would be a monomer and active as a polymerase, and PR would no longer be active. After Pol cleavage, PR-RT has very high levels of reverse transcriptase and RNase H activities [58,59], and free IN is active [39]. After infection of new cells, weak dimers of PR-RT, albeit at a low level in virions, allow PR to cleave at an additional site in Gag, yielding an approximately 38 kD-Gag in newly infected cells [84]. This secondary cleavage of Gag is thought to be required for complete disassembly in newly infected cells. Recently, another report showed that virions produced from cells cotransfected with p68 processed Gag with PR-deficient Pol are infectious, although infectivity is reduced to 0.5-2% of WT [57], suggesting that PR activity is not absolutely essential at an early stage after target cell entry. Overall, it appears that FV PR activity depends on the dimerization of PrPol during the assembly process and is then downregulated after assembly is complete.

## 5. Conclusions

FV Pol is not synthesized as a Gag-Pol protein, as in orthoretroviruses. Rather, it is expressed from a spliced mRNA. Thus, there are unique mechanisms for regulation of Pol expression and packaging. Only Pol precursor protein, PR-RT-IN, is packaged. Protease cleavage of PrPol results in PR-RT and IN proteins, but neither protein by itself is packaged. Pol packaging requires RNA motifs in genomic RNA, called PES (Pol encapsidation sequences). Pol packaging mediated through PES requires both the Pol IN domain, as well as a GR box in the Gag protein. It is likely that a Gag-Pol complex is packaged through binding to the PES, but it remains to be determined how this complex is formed. The FV Pol precursor dimerizes through the IN domain. There may also be a role for an RNA sequence, PARM. All retroviral proteases must dimerize to create an active site. Dimerization of FV PrPol is required for PR activation. FV RT is active as a monomer. This poses a problem for FV, since PR and RT are expressed as a fusion protein. A model is presented to explain this paradox. PrPol has protease activity, which results in cleavage of IN from the precursor. The resultant PR-RT is now monomeric and has RT activity. FV reverse transcription occurs during virus assembly and/or budding, resulting in dsDNA-containing infectious virions. The timing of FV reverse transcription is unique among retroviruses and is independent of the mode of Pol expression. The timing of RT is not the result of the absence of Gag in the precursor. Instead, RT timing appears to be intrinsic to the Pol protein.

## References

[1] Katzourakis A., Gifford R.J., Tristem M., Gilbert M.T., Pybus O.G. (2009). Macroevolution of Complex Retroviruses. Science.

[2] Han G.Z., Worobey M. (2012). An Endogenous Foamy Virus in the Aye-Aye (Daubentonia madagascariensis). J. Virol..

[3] Han G.Z., Worobey M. (2012). An endogenous foamy-like viral element in the coelacanth genome. PLoS Pathog.

[4] Meiering C.D., Linial M.L. (2001). Historical perspective of foamy virus epidemiology and infection. Clin. Microbiol. Rev..

[5] Jones-Engel L., May C.C., Engel G.A., Steinkraus K.A., Schillaci M.A., Fuentes A., Rompis A., Chalise M.K., Aggimarangsee N., Feeroz M.M., Grant R., Allan J.S., Putra A., Wandia N., Watanabe R., Kuller L., Thongsawat S., Chaiwarith R., Kyes R.C., Linial M.L. (2008). Diverse Contexts of Zoonotic Transmission of Simian Foamy Viruses in Asia. Emerg. Infect. Dis..

[6] Mouinga-Ondémé A., Caron M., Nkoghé D., Telfer P., Marx P., Saïb A., Leroy E., Gonzalez J.P., Gessain A., Kazanji M. (2012). Cross-Species Transmission of Simian Foamy Virus to Humans in Rural Gabon, Central Africa. J. Virol..

[7] Linial M.L. (1999). Foamy viruses are unconventional retroviruses. J. Virol..

[8] Leceilier C.-H., Saïb A. (2000). Foamy viruses: between retroviruses and pararetroviruses. Virology.

[9] Goff S.P., Knipe D.M., Howley P.M. Retroviridae: The retroviruses and their replication. Fields Virology.

[10] Peters K., Wiktorowicz T., Heinkelein M., Rethwilm A. (2005). RNA and protein requirements for incorporation of the Pol protein into foamy virus particles. J. Virol..

[11] Yu S.F., Edelmann K., Strong R.K., Moebes A., Rethwilm A., Linial M.L. (1996). The carboxyl terminus of the human foamy virus gag protein contains separable nucleic acid binding and nuclear transport domains. J. Virol..

[12] Schliephake A.W., Rethwilm A. (1994). Nuclear localization of foamy virus Gag precursor protein. J. Virol..

[13] Stenbak C.R., Linial M.L. (2004). Role of the C terminus of foamy virus Gag in RNA packaging and Pol expression. J. Virol..

[14] Lee E.-G., Linial M.L. (2008). The C terminus of foamy retrovirus Gag contains determinants for encapsidation of Pol protein into virions. J. Virol..

[15] Müllers E., Uhlig T., Stirnnagel K., Fiebig U., Zentgraf H., Lindemann D. (2011). Novel Functions of Prototype Foamy Virus Gag Glycine- Arginine-Rich Boxes in Reverse Transcription and Particle Morphogenesis. J. Virol..

[16] Yu S.F., Eastman S.W., Linial M.L. (2006). Foamy virus capsid assembly occurs at a pericentriolar region through a cytoplasmic targeting/retention signal in Gag. Traffic.

[17] Beckham C.J., Parker R. (2008). P bodies, stress granules, and viral life cycles. Cell. Host Microbe..

[18] Furtak V., Mulky A., Rawlings S.A., Kozhaya L., Lee K., KewalRamani V.N., Unutmaz D. (2010). Perturbation of the P-Body Component Mov10 Inhibits HIV-1 Infectivity. PLoS ONE.

[19] Burdick R., Smith J.L., Chaipan C., Friew Y., Chen J., Venkatachari N.J. (2010). Delviks-Frankenberry KA, Hu WS, Pathak VK P Body-Associated Protein Mov10 Inhibits HIV-1 Replication at Multiple Stages. J. Virol..

[20] Jangra R.K., Yi M., Lemon S.M. (2010). DDX6 (Rck/p54) Is Required for Efficient Hepatitis C Virus Replication but Not for Internal Ribosome Entry Site-Directed Translation. J. Virol..

[21] Ariumi Y., Kuroki M., Abe K., Dansako H., Ikeda M., Wakita T., Kato N. (2007). DDX3 DEAD-Box RNA Helicase Is Required for Hepatitis C Virus RNA Replication. J. Virol..

[22] Yu S.F., Lujan P., Jackson D.L., Emerman M., Linial M.L. (2011). The DEAD-box RNA Helicase DDX6 is Required for Efficient Encapsidation of a Retroviral Genome. PLoS Pathog.

[23] Seeger C., Zoulim F., Mason W.S., Knipe D.M., Howley P.M. 2007 Hepadnaviruses. Fields Virology.

[24] Yu S.F., Baldwin D.N., Gwynn S.R., Yendapalli S., Linial M.L. (1996). Human foamy virus replication - a pathway distinct from that of retroviruses and hepadnaviruses. Science.

[25] Jordan I., Enssle J., Guttler E., Mauer B., Rethwilm A. (1996). Expression of human foamy virus reverse transcriptase involves a spliced pol mRNA. Virology.

[26] Löchelt M., Flügel R.M. (1996). The human foamy virus pol gene is expressed as a Pro-Pol polyprotein and not as a Gag-Pol fusion protein. J. Virol..

[27] Bodem J., Löchelt M., Winkler I., Flower R.P., Delius H., Flügel R.M. (1996). Characterization of the spliced *pol* transcript of feline foamy virus - the splice acceptor site of the *pol* transcript is located in *gag* of foamy viruses. J. Virol..

[28] Holzschu D.L., Delaney M.A., Renshaw R.W., Casey J.W. (1998). The nucleotide sequence and spliced pol mRNA levels of the nonprimate spumavirus bovine foamy virus. J. Virol..

[29] Lee E.-G., Kuppers D., Horn M., Roy J., May C., Linial M.L. (2008). A Premature Termination Codon Mutation at the C Terminus of Foamy Virus Gag Downregulates the Levels of Spliced pol mRNA. J. Virol..

[30] Baldwin D.N., Linial M.L. (1998). The roles of Pol and Env in the assembly pathway of human foamy virus. J. Virol..

[31] Heinkelein M., Leurs C., Rammling M., Peters K., Hanenberg H., Rethwilm A. (2002). Pregenomic RNA is required for efficient incorporation of Pol polyprotein into foamy virus capsids. J. Virol..

[32] Heinkelein M., Schmidt M., Fischer N., Moebes A., Lindemann D., Enssle J., Rethwilm A. (1998). Characterization of a *cis*-acting sequence in the *pol* region required to transfer human foamy virus vectors. J. Virol..

[33] Erlwein O., Bieniasz P.D., McClure M.O. (1998). Sequences in *pol* are required for transfer of human foamy virus- based vectors. J. Virol..

[34] Trobridge G., Josephson N., Vassilopoulos G., Mac J., Russell D.W. (2002). Improved foamy virus vectors with minimal viral sequences. J. Am. Soc. Gene Ther..

[35] Heinkelein M., Dressler M., Jarmy G., Rammling M., Imrich H., Thurow J., Lindemann D., Rethwilm A. (2002). Improved primate foamy virus vectors and packaging constructs. J. Virol..

[36] Lee E.-G., Sinicrope A., Jackson D.L., Yu S.F., Linial M.L. (2012). Foamy Virus Pol Protein Expressed as a Gag-Pol Fusion Retains Enzymatic Activities, Allowing for Infectious Virus Production. J. Virol..

[37] Müllers E., Stirnnagel K., Kaulfuss S., Lindemann D. (2011). Prototype Foamy Virus Gag Nuclear Localization: a Novel Pathway among Retroviruses. J. Virol..

[38] Cartellieri M., Rudolph W., Herchenroder O., Lindemann D., Rethwilm A. (2005). Determination of the relative amounts of Gag and Pol proteins in foamy virus particles. Retrovirol..

[39] Roy J., Linial M.L. (2007). Role of the foamy virus Pol cleavage site in viral replication. J. Virol..

[40] Lee E.-G., Roy J., Jackson D., Clark P., Boyer P.L., Hughes S.H., Linial M.L. (2011). Foamy Retrovirus Integrase Contains a Pol Dimerization Domain Required for Protease Activation. J. Virol..

[41] Konvalinka J., Löchelt M., Zentgraf H., Flügel R.M., Krausslich H.-G. (1995). Active spumavirus proteinase is essential for virus infectivity but not for formation of the Pol polyprotein. J. Virol..

[42] Enssle J., Fischer N., Moebes A., Mauer B., Smola U., Rethwilm A. (1997). Carboxy-terminal cleavage of the human foamy virus gag precursor molecule is an essential step in the viral life cycle. J. Virol..

[43] Zemba M., Wilk T., Rütten T., Wagner A., Flügel R.M., Löchelt M. (1998). The carboxy-terminal p3(Gag) Domain of the human foamy virus gag precursor is required for efficient virus infectivity. Virology.

[44] Pearl L.H., Taylor W.R. (1987). A structural model for the retroviral proteases. Nature.

[45] Wlodawer A., Miller M., Jaskolski M., Sathyanarayana B.K., Baldwin E., Weber I.T., Selk L.M., Clawson L., Schneider J., Kent S.B. (1989). Conserved folding in retroviral proteases: crystal structure of a synthetic HIV-1 protease. Science.

[46] Miller M., Jaskolski M., Rao J.K.M., Leis J., Wlodawer A. (1989). Crystal structure of a retroviral protease proves relationship to aspartic protease family. Nature.

[47] Boross P., Tozser J., Bagossi P. (2006). Improved purification protocol for wild-type and mutant human foamy virus proteases. Prot. Exp. Purific..

[48] Pfrepper K.I., Rackwitz H.R., Schnolzer M., Heid H., Löchelt M., Flügel R.M. (1998). Molecular characterization of proteolytic processing of the Pol proteins of human foamy virus reveals novel features of the viral protease. J. Virol..

[49] Hartl M.J., Wöhrl B.M., Rosch P., Schweimer K. (2008). The Solution Structure of the Simian Foamy Virus Protease Reveals a Monomeric Protein. J. Mol. Biol..

[50] Hartl M., Mayr F., Rethwilm A., Wöhrl B. (2010). Biophysical and enzymatic properties of the simian and prototype foamy virus reverse transcriptases. Retrovirol..

[51] Hartl M.J., Schweimer K., Reger M.H., Schwarzinger S., Bodem J., Rοsch P., Wöhrl B.M. (2010). Formation of transient dimers by a retroviral protease. Biochem. J..

[52] Li M., Mizuuchi M., Burke T.R., Craigie R. (2006). Retroviral DNA integration: reaction pathway and critical intermediates. EMBO J..

[53] Hare S., Gupta S.S., Valkov E., Engelman A., Cherepanov P. (2010). Retroviral intasome assembly and inhibition of DNA strand transfer. Nature.

[54] Gupta K., Curtis J., Krueger S., Hwang Y., Cherepanov P., Bushman F., Van-áDuyne G. (2012). Solution Conformations of Prototype Foamy Virus Integrase and Its Stable Synaptic Complex with U5 Viral DNA. Structure.

[55] Hartl M.J., Bodem J., Jochheim F., Rethwilm A., Rosch P., Wöhrl B.M. (2011). Regulation of Foamy Virus Protease Activity by Viral RNA: a Novel and Unique Mechanism among Retroviruses. J. Virol..

[56] Spannaus R., Hartl M., Wöhrl B., Rethwilm A., Bodem J. (2012). The prototype foamy virus protease is active independently of the integrase domain. Retrovirol..

[57] Hütter S., Müllers E., Stanke N., Reh J., Lindemann D. (2013). Prototype Foamy Virus protease activity is essential for intra-particle reverse transcription initiation but not absolutely required for uncoating upon host cell entry. J. Virol..

[58] Rinke C.S., Boyer P.L., Sullivan M.D., Hughes S.H., Linial M.L. (2002). Mutation of the catalytic domain of the foamy virus reverse transcriptase leads to loss of processivity and infectivity. J. Virol..

[59] Boyer P.L., Stenbak C.R., Clark P.K., Linial M.L., Hughes S.H. (2004). Characterization of the polymerase and RNase H activities of human foamy virus reverse transcriptase. J. Virol..

[60] Santos-Velazquez J., Kim B. (2008). Deoxynucleoside triphosphate incorporation mechanism of foamy virus (FV) reverse transcriptase: implications for cell tropism of FV. J. Virol..

[61] Swiersy A., Wiek C., Reh J., Zentgraf H., Lindemann D. (2011). Orthoretroviral-like Prototype Foamy Virus Gag-Pol expression is compatible with viral replication. Retrovirol..

[62] Nassal M. (2008). Hepatitis B viruses: reverse transcription a different way. Virus. Res..

[63] Wang G.H., Seeger C. (1993). Novel mechanism for reverse transcription in hepatitis B viruses. J. Virol..

[64] Delelis O., Saïb A., Sonigo P. (2003). Biphasic DNA Synthesis in Spumaviruses. J. Virol..

[65] Zamborlini A., Renault N.M., Saïb A., Delelis O. (2010). Early Reverse Transcription Is Essential for Productive Foamy Virus Infection. PLoS ONE.

[66] Yu S.F., Sullivan M.D., Linial M.L. (1999). Evidence that the human foamy virus genome is DNA. J. Virol..

[67] Moebes A., Enssle J., Bieniasz P.D., Heinkelein M., Lindemann D., Bock D., McClure M.O., Rethwilm A. (1997). Human foamy virus reverse transcription that occurs late in the viral replication cycle. J. Virol..

[68] Jackson D.L., Lee E.-G., Linial M.L. (2013). Expression of Prototype Foamy Virus Pol as a Gag-Pol Fusion Protein Does Not Change the Timing of Reverse Transcription. J. Virol..

[69] Pahl A., Flügel R.M. (1995). Characterization of the human spumaretrovirus integrase by site- directed mutagenesis, by complementation analysis, and by swapping the zinc finger domain of HIV-1. J. Biol. Chem..

[70] Lee H.S., Kang S.Y., Shin C.-G. (2005). Characterization of the functional domains of human foamy virus integrase using chimeric integrases. Mol. & Cells.

[71] Imrich H., Heinkelein M., Herchenroder O., Rethwilm A. (2000). Primate foamy virus Pol proteins are imported into the nucleus. J. Gen. Virol..

[72] An D.G., Hyun U., Shin C.G. (2008). Characterization of nuclear localization signals of the prototype foamy virus integrase. J. Gen. Virol..

[73] Fouchier R.A., Malim M.H. (1999). Nuclear import of human immunodeficiency virus type-1 preintegration complexes. Adv. Vir. Res..

[74] Lo Y.T., Tian T., Nadeau P.E., Park J., Mergia A. (2010). The foamy virus genome remains unintegrated in the nuclei of G1/S phase-arrested cells, and integrase is critical for preintegration comlex transport into the nucleus. J. Virol..

[75] Maertens G.N., Hare S., Cherepanov P. (2010). The mechanism of retroviral integration from X-ray structures of its key intermediates. Nature.

[76] Delelis O., Carayon K., Guiot E., Leh H., Tauc P., Brochon J.C., Mouscadet J.F., Deprez E. (2008). Insight into the Integrase-DNA Recognition Mechanism: A specific DNA-binding mode revealed by an enzymatically labeled integrase. J. Biol. Chem..

[77] Valkov E., Gupta S.S., Hare S., Helander A., Roversi P., McClure M., Cherepanov P. (2009). Functional and structural characterization of the integrase from the prototype foamy virus. Nucleic Acids Res..

[78] Poeschla E.M. (2008). Integrase, LEDGF/p75 and HIV replication. Cell Mol. Life Sci..

[79] Tang C., Louis J.M., Aniana A., Suh J.Y., Clore G.M. (2008). Visualizing transient events in amino-terminal autoprocessing of HIV-1 protease. Nature.

[80] Ishima R., Torchia D.A., Lynch S.M., Gronenborn A.M., Louis J.M. (2003). Solution Structure of the Mature HIV-1 Protease Monomer: Insight into the tertiary fold and stability of a precursor. J. Biol. Chem..

[81] Wondrak E.M., Nashed N.T., Haber M.T., Jerina D.M., Louis J.M. (1996). A Transient Precursor of the HIV-1 Protease. J. Biol. Chem..

[82] Life R.B., Lee E.-G., Eastman S.W., Linial M.L. (2008). Mutations in the amino-terminus of foamy virus Gag disrupt morphology and infectivity, but not targeting of assembly. J. Virol..

[83] Eastman S.W., Linial M.L. (2001). Identification of a conserved residue of foamy virus Gag required for intracellular capsid assembly. J. Virol..

[84] Lehmann-Che J., Giron M.L., Delelis O., Lochelt M., Bittoun P., Tobaly-Tapiero J., de The H., Saib A. (2005). Protease-dependent uncoating of a complex retrovirus. J. Virol..

